# Kickboxing therapy in a Day hospital for non-psychotic disorders

**DOI:** 10.1192/j.eurpsy.2025.1597

**Published:** 2025-08-26

**Authors:** M. Krevatin, S. Kocijan, H. Matic, A. Jurcevic, E. Ivezic, A. Ivrlac, S. Caratan, L. Gorsic, M. Horvat, M. Rimac

**Affiliations:** 1 Day Hospital for non psychotic disorders, University Psychiatric Hospita Sveti Ivan, Zagreb; 2 Department of Psychiatry and Neurology, Faculty of Dental Medicine J.J.Strossmayer and Health, Osijek, Croatia

## Abstract

**Introduction:**

The limited research conducted so far suggests that kickboxing and other martial arts can positively impact psychological well-being in a psychotherapeutic setting. The study by de Vries et al. (de Vries, et al.), which focuses on the application of kickboxing with psychotic patients, suggests improvements in self-esteem, social behavior, and aggression regulation through structured, body-oriented therapy. Physical activity that is intense and structured, such as kickboxing, helps reduce stress and anxiety by increasing endorphin production, which improves mood and reduces tension.

**Objectives:**

The aim of our study is to analyze the impact of kickboxing on non-psychotic disorders in relation to anxiety, depression, and stress levels, as well as its effect on self-esteem and the overall quality and satisfaction. To our knowledge, this research is the first of its kind in Croatia.

**Methods:**

This study involved 11 participants who were receiving treatment at the Day Hospital for Non-Psychotic Disorders, which treats patients suffering from a wide range of anxiety disorders, mood disorders, reactions to severe stress, and adjustment disorders. The participants were a mixed group of 6 men and 5 women, aged 28 to 58, with varying levels of education and living standards. Kickboxing training has been conducted once a week for 60 minutes during consecutive 6 months. The training is conducted in a group with an individualized approach depending on the patient’s condition. The assessment was conducted at two time points: the first data collection occurred before the start of therapy, and the second took place 6 months after regular kickboxing training. The comparison was made using the Wilcoxon signed-rank test.

**Results:**

The results of this preliminary study indicate statistically significant positive changes. Compared to the results of the first assessment, participants were significantly more satisfied with their lives in the second assessment and perceived their quality of life as significantly improved in terms of physical and mental health as well as social relationships. Depression, anxiety, and stress levels were rated as significantly less pronounced, while self-liking and self-competence did not change significantly.

**Conclusions:**

Kickboxing offers a new, innovative approach to improving mental health in the therapeutic environment of a day hospital, especially for patients suffering from anxiety and depression.

**Disclosure of Interest:**

None Declared

